# SDN-based Handover Scheme in Cellular/IEEE 802.11p Hybrid Vehicular Networks †

**DOI:** 10.3390/s20041082

**Published:** 2020-02-17

**Authors:** Ran Duo, Celimuge Wu, Tsutomu Yoshinaga, Jiefang Zhang, Yusheng Ji

**Affiliations:** 1 Graduate School of Informatics and Engineering, The University of Electro-Communications, Tokyo 1828585, Japan; duoran@comp.is.uec.ac.jp (R.D.); yoshinaga@uec.ac.jp (T.Y.); 2 Institute of Intelligent Media Technology, Communication University of Zhejiang, Hangzhou 310018, China; 20100937@cuz.edu.cn; 3 Information Systems Architecture Research Division, National Institute of Informatics, Tokyo 1018430, Japan; kei@nii.ac.jp

**Keywords:** VANETs, SDN-based handover, Mobile edge computing, Cellular/IEEE 802.11p hybrid vehicular networks

## Abstract

With the arrival of 5G, the wireless network will be provided with abundant spectrum resources, massive data transmissions and low latency communications, which makes Vehicle-to-Everything applications possible. However, VANETs always accompany with frequent network topology changes due to the highly mobile feature of vehicles. As a result, the network performance will be affected by the frequent handover. In this paper, a seamless handover scheme is proposed where the Software-Defined Networking (SDN) and Mobile Edge Computing (MEC) technologies are employed to adapt to the dynamic topology change in VANETs. The introduction of SDN provides a global view of network topology and centralized control, which enables a stable transmission layer connection when a handover takes place, so that the upper layer performance is not influenced by the network changes. By employing MEC server, the data are cached in advance before a handover happens, so that the vehicle can restore normal communication faster. In order to confirm the superiority of our proposal, computer simulations are conducted from different aspects. The results show that our proposal can significantly improve the network performance when a handover happens.

## 1. Introduction

In recent years, with the rapid development of communication technology, the Vehicular Ad hoc NETwork (VANET) technology [1,2] is regarded as one of the main solutions that are able to provide a wide variety of services in vehicular environments. In VANETs, vehicles can act as network nodes and communicate with each other to build up a large network. VANETs naturally inherit basic characteristics of Mobile Ad hoc NETworks (MANETs), but possess special features, such as the high mobility and varying network densities. The future fifth generation (5G) network [3,4] is supposed to provide high reliability and low latency communications. The arrival of 5G greatly promotes the development of Internet of Things (IoT) technology. The vehicular to everything (V2X) communication, is considered as the one of key use cases in future 5G system. To support the future 5G-based V2X application such as automated driving and overtake, VANETs need smarter, faster and more reliable communications to meet Ultra-Reliable Low-latency requirement [5]. In VANETs, vehicles move in a high speed, and therefore the network topology will face highly dynamic changes. In most cases, vehicles would get network services through roadside infrastructures. For the vehicle-to-roadside communications, the velocity variation of vehicles affects their location which in turn causes a frequent handover between base stations and different roadside units (RSU). A handover is always accompanied by the connection change, such as change of IP address, which makes it difficult to have a stable wireless connection especially at the transport layer. The performance of the network will be effected by the frequent handover. Therefore, a design of efficient handover protocol becomes particularly important to meet the high traffic load, low latency and high reliability requirements of mobile services.

Internet Engineering Task Force community released mobility support protocols in the network layer [6,7] and RFC 3963 [8] to support the wireless mobility and handoff management respectively. By taking actions after the occurrence of handover, these approaches cannot adapt to the rapidly changing network topology in VANETs. Reference [9] uses a cluster head centric network topology to reduce the handover frequency by a hierarchical architecture where only the cluster head nodes are connected to the RSU. Although this approach could achieve a lower overhead by reducing the number of nodes involved in the handover process, but the data losses still exist when the cluster head handover to the other RSUs, and the vehicles in the cluster still face the performance degradation problem. Therefore, we propose a handover scheme to adapt frequent network change and clustered vehicular network topology.

As more and more data are generated by the mobile devices, a new concept Mobile Edge Computing (MEC) emerges. In contrast to the cloud computing, which is more centralized, MEC is deployed at the edge of the mobile network, that is, near to the mobile node. Since the edge is localized, it can immediately respond to the changes in the surrounding environment to support the need of low latency and location-aware services, so the MEC is used as an effective way in offloading. For example, Reference [10] introduces MEC to offload the traffic and computation from the vehicles which achieves reduction of service delay and energy consumption. In order to better support the traffic offload at the edge, Reference [11] considers the influence of transmission latency and storage cost to enhance the quality of experience of caching. In this paper, MEC is employed to support handover by proactively caches the data at the new base station. Another key technology worth mentioning is the Software-Defined Networking (SDN). SDN divides the network into a control layer and a data layer, thereby separating the control decisions of the network from the forwarding actions. With the flexibility, programmability and centralized control feature of SDN, the deployment of VANETs services become more flexible, efficient and secure. In this paper, we propose a scheme that uses SDN technology to achieve a global view of network and conducts a handover by maintaining the same address.

The paper is an extension of our previous conference paper [12] where an efficient handoff approach for SDN-based IEEE 802.11p/LTE VANETs is discussed. In this paper, we improve the previous work by proposing a new caching algorithm based on mobile edge computing, and also introduce a new idea of two level SDN controller that utilizes the global view of SDN to improve the overall efficiency. The contributions of this paper are two folds:

- SDN-based handover approach: we propose a two-level SDN-based architecture, where central SDN controller keeps monitoring the network topology and produces a global view of the network, and the edge SDN controllers gather vehicle information and report to central controller, as well as deploy specific actions to the vehicles. The handover approach is discussed from two different aspects to ensure handover integrity.
- Data caching on MEC server: we introduce a MEC server on the base station to support caching scheme, so as to guarantee the data transmissions. The data under transmissions will be cached on the MEC server which belongs to the another base station that the vehicle will handover to. The data caching happens when a handover happens between two base stations.

The rest of the paper is organized as follows. We summarize the related work in Section 2, and the architecture of the proposed scheme is described in Section 3. The proposed data caching approach is explained in Section 4. Section 5 describes the details of the vehicular handover procedure in the proposed scheme. We demonstrate simulation results in Section 6, before concluding this paper in Section 7.

## 2. Related Work

### 2.1. Handover in the Networks with Low Mobility

To support the network-layers handover, Reference [6] stipulates the Mobile IP protocol that allows a mobile node to be equipped by both home address and care-of address and achieves datagrams delivery in different subnetworks by passing through a tunnel between home agent and foreign agent. As well as this, Reference [7] defines a new IPv6 protocol and option to support mobility in IPv6 network, by which the mobile node could directly receive the packages through its care-of address. In order to enable handover in hybrid networks, Reference [13] investigates the mobile node’s handover in mixed IPv4/IPv6 environment, and provides handover procedure for different scenarios. Besides, Reference [14] proposes handover decision using a Kalman filter and fuzzy logic in heterogeneous wireless networks and shows handover decision from cellular networks to WLAN. Reference [15] proposes a design in wireless mesh networks, which separates the backhaul channels into data packets and signaling packets to reduce handover latency. In Reference [16], a preemptive handoff strategy is proposed to maintain reliable links by exploiting channel state information. Although the above studies conceive to support the network with mobile nodes, they do not consider a network with frequent handover.

### 2.2. Handover in VANETs

There has been a large number of seamless handover approaches. For example, in Reference [17], NEMO and VANETs are combined to achieve a seamless handover between different access points. When the handover is about to happen, the vehicle can get the available information of new access point in advance through assistance from other vehicles, so that the new access point can be configured immediately according to the need of vehicle, reducing handover latency. In some studies, VANETs are divided into different clusters to support scalability and reliability. For the clustered VANETs, the handover happens not only between different access points, but also between different clusters and optimized by introducing a cluster head in each cluster [18]. For example, Reference [19] collects drivers’ behaviors and predicts the speed and position of cluster members to select the second cluster head as a backup, which indicates the cluster may handover to, but the handover of cluster head change between the base stations is not discussed. Reference [20] maximizes the connection time between the vehicles by introducing vehicular link expiration time metric considering road topology and the possibility of vehicles movement, to minimize the handover frequency. From the security perspective, handover process needs to be authenticated by the new access point, so that Reference [21] develops a kind of clustering algorithm based on relative velocity, position and signal strength, and proposes a group authentication scheme on 5G environment to decrease handover authentication computing overhead. From the aspect of radio spectrum handover, Reference [22] employs the hidden Markov model to estimate channel state and proactively predicts the handover. Besides, it picks up the most important lightweight information from the data and sends them to the destination before the handover to hide the handover occurrence from the user perception. As IEEE 802.11p is the main standard for vehicular communications, its poor scalability, low capacity and intermittent connectivity problems have to be solved. Reference [23] considers the Long Term Evolution (LTE) mobile communication technologies to support vehicular applications. Reference [24] proposes a novel vertical handover protocol for seamless switching between IEEE 802.11p and LTE networks. However, these proposals do not consider the influence of handover from the view of the transport layer. Reference [25] considers the fact that TCP connection disruption could occur when handover happens between vehicles and roadside units, and proposes a beacons control data dissemination protocol. It enables the vehicle to disconnect and reconnect TCP communication actively when a handover happens between RSUs to reduce the effects from TCP connection disruption on the network performance during the handover process. Although the performance at the transport layer is considered in this proposal, but the connection disruption still exists during the handover process.

### 2.3. SDN-based VANETs

As SDN gives revolutionary thinking to the network, researchers are attracted by the SDN-based VANETs architecture. Reference [26] proposes a kind of network that applies the SDN concept to VANETs to achieve network programmability and flexibility, and improve the management of mobile devices and resources. Besides, Reference [26] explains that SDN can be applied to safety surveillance and virtualization services. As the SDN controller requires to centralize global knowledge and configure the network, Reference [27] proposes a scalable and dynamic access control scheme to ensure the security of north bound interface. In order to support the cloud computing at the edge of network, Reference [28] proposes a networking architecture in which not only SDN technology is used but also fog computing is added to provide security services, traffic management, delay reduction and location aware functions. As an important technology in 5G network, SDN provides solutions for low power consumption, efficient resource management, and better scalability [29,30]. A three-plane software-defined 5G architecture that additionally introduces energy plane to the network, is first presented in Reference [31] to control energy consumption by using SDN to monitor data flows. A 5G-based software defined vehicular network architecture is proposed in [32]. Besides, Reference [32] utilizes the natural function of SDN in information collection and network management to achieve adaptive vehicle clustering, and proposes a dual CH design to enhance the network robustness. Furthermore, An all-SDN network architecture is proposed in Reference [33]. This architecture, on the one hand, introduces hierarchical controllers to offer flow-based service and offers unified handover and routing decisions. On the other hand, it discusses programmable handover occurrences between different devices. Considering the increasing number of vehicles, Reference [34] uses SDN to provide rational use of resources. It regards the group of parking vehicles as fog computing infrastructures and proposes SDN-based multi-level architecture. Reference [35] investigates the security and privacy issue using block chain in the transportation system and the vehicular IoT environment in SDN-enabled 5G-VANETs. To make use of the centralized function advantage of the SDN, a social-aware cluster algorithm is proposed in Reference [36] to model the vehicle movement in predicting the future routes. Although, SDN-based handover is mentioned in some studies, but they do not specify the handover process in detail. In this paper, we use SDN to achieve high performance handover in VANETs.

## 3. Proposed SDN-based VANET Architecture

We manage vehicular networks with two-level SDN controller architecture. The level 1 is composed of SDN central controller connecting with the core network. Level 1 could have a global view of the network and execute clustering only when it is needed. Level 2 is formed by base stations which are equipped with SDN controllers and MEC servers to proactively cache data in MEC servers to reduce packet loss during the handover.

The wireless network is built based on two kinds of wireless spectrums. For the communications among vehicles, the network architecture uses IEEE 802.11p to support large amount of data traffics in a high-speed mobile environment. Besides, for the communication between a vehicle and a base station, cellular network (such as LTE and 5G network) is used to support long range wireless connections [37]. IEEE 802.11p-based VANETs utilize unlicensed bands to provide communications for vehicle-to-everything. A vehicle could be connected to Internet either through direct cellular network or through another vehicle which has connection to cellular network. For VANETs, a vehicle with cellular connection works as a gateway using the cellular network to provide communication services. The communication parameters between vehicles are set based on the IEEE 802.11p specification, working at the frequency of 5.9 GHz with 7 channels and providing about 300 m communication distance with the data rate ranging from 6 Mbps to 27 Mbps.

The paper introduces SDN in the network architecture to realize the coordinated control and information exchange between network equipment. The main idea of SDN is to separate the control plane and data plane in the network. SDN enabled devices process packets according to the specific established policies which are distributed or modified by the SDN controller. In vehicular networks, SDN removes the control function from the basic infrastructure to the control plane so that applications can be implemented on the SDN controller to achieve various functions including the monitoring, traffic control and cluster management [38]. In this way, SDN improves the management of both resources and vehicles, and creates a great opportunity for new services and control functions.

**Figure 1:**
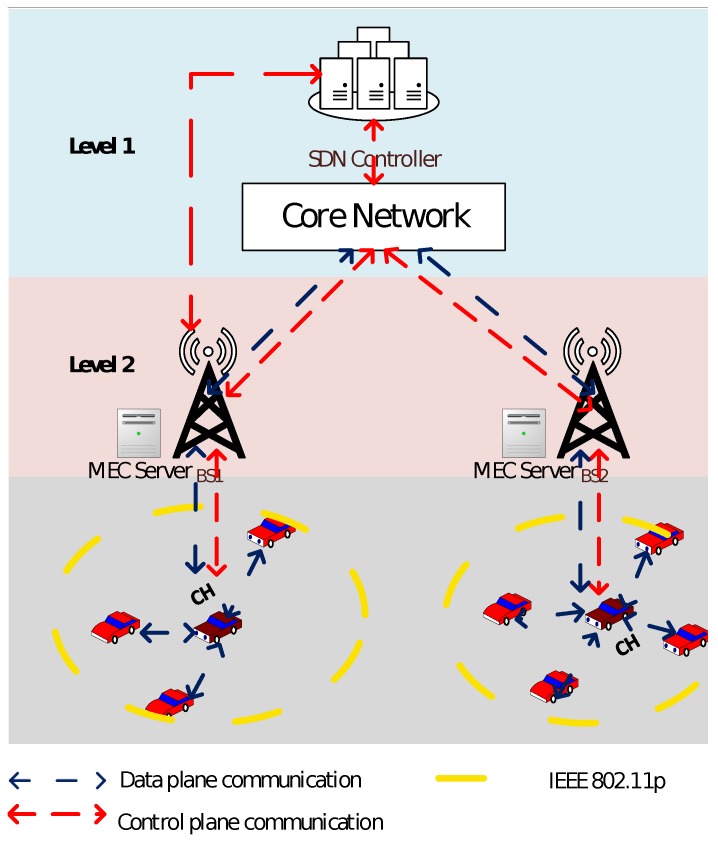
Software defined networking (SDN) enabled 802.11p/cellular hybrid Vehicular Ad hoc NETwork (VANET) architecture.

As shown in Figure 1, vehicles are divided into several clusters. Each vehicle in the network is a SDN enabled device and equipped with both cellular interface and IEEE 802.11p interface. Vehicles in the same cluster use IEEE 802.11p protocol to get VANET service by communicating with each other, which saves scarce spectrum resources and mitigates the burden on the cellular. Besides, a Cluster Head (CH) communicates with a cluster member by IEEE 802.11p and with an base station by LTE [39]. Vehicles that work as the cluster members connect with an base station through the CH to get service, so that the signal overhead could be reduced during the handover process.

## 4. MEC Deployment

In the conventional approach, when the handover happens, the data being transmitted through old base stations cannot be completely received by the vehicle. As a result, data are lost without being noticed by the server and vehicle. Therefore, in order to get complete data, the vehicle sends request for the lost data, and on the contrary, more new data will be continuously sent by the server. According to the transport layer’s congestion control, the server will not respond until it receives multiple requests for the same data from the vehicle or a wait timeout appears. As a result, the vehicle cannot get the desired data until a period of delay occurs.

**Figure 2:**
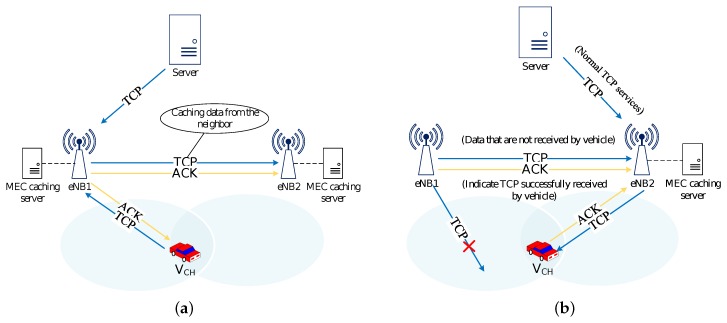
Caching scheme in handover process (**a**) eNB2 starts caching the data before handover; (**b**) eNB2 starts communications with vehicle.

In order to overcome the described problem, we introduce the concept of MEC [40] in our handover approach. We deploy one MEC server at each base station between the access network and core network. The MEC server can cache the data from the server and reduce the time cost of restoring normal transmission, so that to achieve seamless communication when the handover happens. The Figure 2 shows how does the MEC caching scheme work to support the vehicle handover from eNB1 to eNB2.

As shown in Figure 2a, when indicated to execute handover process, eNB2 starts a caching action to copy the data from the server. The vehicle communicates with server still through eNB1 at this time. And not only the data from the server, but also the ACK messages from the vehicle are duplicated to the eNB2. According to the information of received ACK which carries the data requirement of vehicle, MEC server will release the unnecessary data from the cache.

Figure 2b shows when the vehicle is ready to communicate with server through eNB2, the data will be transmitted from the eNB2 MEC server that have already been cached. If ACK from vehicle is received first, eNB2 responds to the vehicle with the required data. Otherwise, eNB2 sends a segment requesting for the ACK to confirm the data sequence to be sent.

To support caching scheme, we maintain a caching queue for each service on the base station cache. The MEC module then caches the unreceived data and drops the received data by analyzing the data information to keep the queue at a proper size. Thus, the data are enable to be transmitted without loss and be more correctly to minimize the extra transmissions. We introduce three additional variables per service at a MEC module:

(1) first_cached_seq–stores the first sequence number in the caching queue.
(2) seq_acked–represents the sequence that have been received by the vehicle.
(3) last_cached_seq–stores the last sequence number in the caching queue.

Algorithm 1 shows the caching algorithm at base station. The MEC server continuously collect data from the network, if the current data packet for transmission bounds for the handover vehicle, the algorithm compares the data sequence number with the variables of queues to determine whether to cache the data, and then updates the variables. Besides, if the data packet is from the vehicle, the caching algorithm drops the acknowledged data from the queue according to the ACK field that denotes the next expected data, and then updates the caching queue variables.
**Algorithm 1** Caching algorithm at base station**Initialize:**first_cached_seq=0, seq_acked=0, last_cached_seq=01:Receive packet *i*2:**for** Every packet *i* from wired network **do**3:    **if** Destination address == vehicle’s ID **then**4:        **if** first_cached_seq=0 **then**5:           Cache the package.6:           first_cached_seq=$seq_{i}$7:        **else if** seq<first_cached_seq **then**8:           first_cached_seq=$seq_{i}$9:           Cache the package.10:        **else**11:           Drop the package.12:        **end if**13:        **if** last_cached_seq=0 **then**14:           first_cached_seq=$seq_{i}$15:        **else if** last_cached_seq<$seq_{i}$ **then**16:           last_cached_seq=$seq_{i}$17:           Cache the package.18:        **else**19:           Drop the package.20:        **end if**21:    **end if**22:    **if** Source address == vehicle’s ID **then**23:        **if** seq_acked=0 **then**24:        **else if** seq_acked <$ack_{i}$ **then**25:           seq_acked=$ack_{i}$26:        **end if**27:        **if** first_cached_seq<seq_acked **then**28:           Release the caching package from first_cached_seq to seq_acked-129:           first_cached_seq = seq_acked30:        **end if**31:    **end if**32:**end for**

With the use of mobile edge computing in the control of data caching in our proposal, the data loss during the handover can be reduced, and the delay required to resume the normal network communication can be minimized.

## 5. Handover Process Based on SDN

When a handover happens between the clusters, the network address of a vehicle has to be changed to adapt the traffic route change [41]. In this case, for the connection is identified by the network address and port, the transport layer’s connection of service is released and reestablished. As a result, influenced by the congestion window limitation, a degradation of network performance could appear when a handover occurs.

To avoid this result, we introduce SDN to keep the transport layer connection unchanged, achieving a seamless handover. Since SDN can provide a way to allocate and control the network efficiently, it has advantages over traditional approaches in terms of network scalability and transmission efficiency [42]. These advantages could overcome the challenges, such as node mobility, network dynamic characteristic and large network scale. The SDN controller issues the mapping and reverse mapping instruction for the newly accessed vehicle.CH is responsible for the access of the vehicle to the cluster in packet address mapping.

- SDN controller in the core network always monitors the movement of vehicles and cluster information to control the vehicular network. When finding a vehicle is possible to handover to a new cluster, the controller will inform the base station that there could be a handover between two neighboring cluster heads.
- Then, controller on base station notices the new CH about new join in and issues an instruction in advance indicating new mapping rules.
- The new CH receives the instruction and sets the corresponding action with timeout that represents the mapping relationship of a vehicle address to its new address.
- If vehicle does not join the new cluster, the action will be deleted automatically.
- When a vehicle happens a handover to the new cluster, the vehicle could transmit data packet immediately without rerouting computation and communication reconnection. The source address of the transmitting packet could be mapped to the address that is used to indicate the vehicle’s position according to the action set by SDN controller.
- After the handover process, the SDN controller updates the network topology information and waits for the next change.

**Figure 3:**
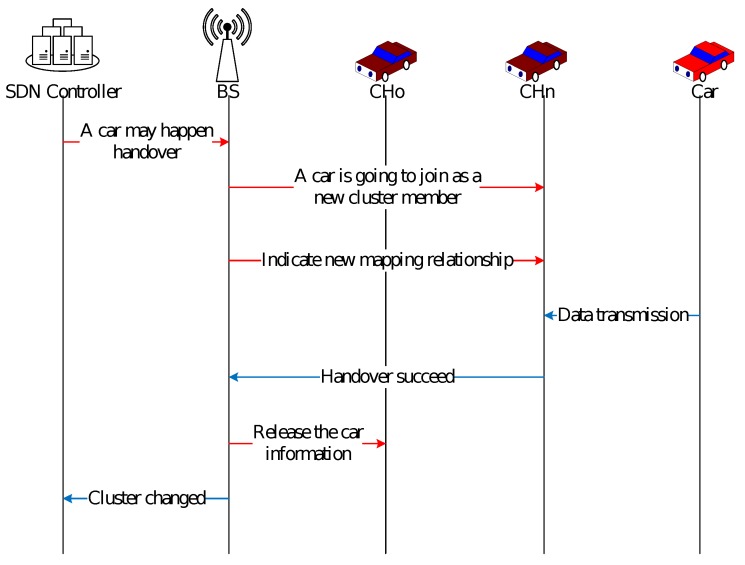
Action setting in advance.

As Figure 3 shows, after discovering a new cluster is possible to join in, the instruction can be distributed by the SDN controller in advance. The controller could distribute instructions as actions to the new cluster head according to the vehicle movement and the status information of the new cluster. The instruction carries a timeout. When the handover does not happen in the new cluster for a long time, the instruction could be automatically deleted. In this way, when joining in a new cluster, vehicle can set up the communication immediately, avoiding the consumption of handover computing time and control information transmission time.

**Figure 4:**
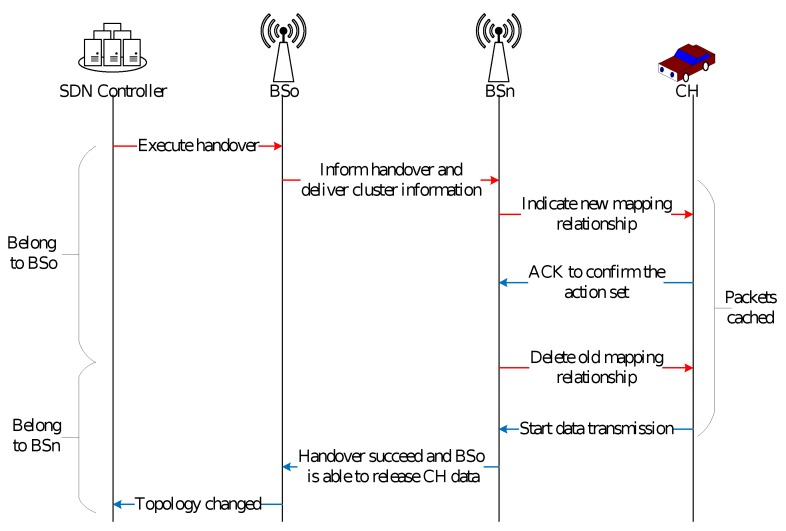
The handover process.

On the other hand, when a handover happens between base stations, a cluster head will execute the series of process as shown in Figure 4.

- When SDN controller finds a cluster is possible to handover between different base stations, it will inform the old base station to execute handover to a new base station.
- Then the old base station informs the new base station of handover and deliver information of cluster preparing for handover.
- The new base station starts to cache the data needed by new cluster and sets the corresponding action with a new mapping relationship of the cluster depending on the information received from the old base station.
- After succeeding in setting action, the old mapping relationship is deleted, so that the data transmission can be proceeded through new base station.
- After the success of handover, the old base station releases the cluster information and informs the SDN controller of the topology changes.

## 6. Simulation Results

### 6.1. Simulation Settings

The simulation is performed in OMNET+5.0 simulator with INET open-source model to compare the performance of our proposal with the conventional method. The vehicle mobility is generated by the SUMO mobility simulator. The Table 1 shows the simulation parameters. We use AODV, a well-known routing protocol for MANETs, as the network routing protocol. However, the AODV protocol makes long delay in the route discovery, which will degrade the network performance. In order to get an accurate evaluation of our proposal, we set “Hello Interval” and “Allowed Hello Loss” in AODV to the minimum size. We conduct simulations in two different road types, namely, grid topology and straight road. The grid topology spans an area of 1000 m × 600 m, with each road segment of 200 m. Node density varied from 180 to 540 nodes in the simulation. In our simulation, we select a vehicle to run a continuous TCP service, connecting to the server in the core network. Each simulation result is the average of 10 runs with different moving routes.

At the transport layer, the data transmissions are based on TCP with the use of RENO congestion control algorithm. In the congestion control algorithm, there is an extra variable, Congestion WiNDow (CWND), tracked to control the TCP traffic rate sending into the network. CWND is initialized when the connection is established, and grows through the congestion control algorithm. If the transport layer connection is interrupted by handover, the CWND will fall into the initial value and then grow through slow start algorithm. We assume that handover always happen between the clusters. The simulation compares our proposal with “Conventional method” (no global view of the network, and no cache is conducted at base station), and “No cache” (with global view but no cache is conducted at base station).

### 6.2. Effect of Data Rates

In the first scenario, we make vehicles happen handover by moving vehicles between different clusters. In order to evaluate how the transmission rate affects the network performance with occurrence of handover, we set different link rates of 3 Mbps, 6 Mbps, 9 Mbps and 12 Mbps for our proposal and change connection handover method (conventional) when running the simulation. Each vehicle moves at the speed of 60 Km/h and happens handover when moving across the different base stations.

**Figure 5:**
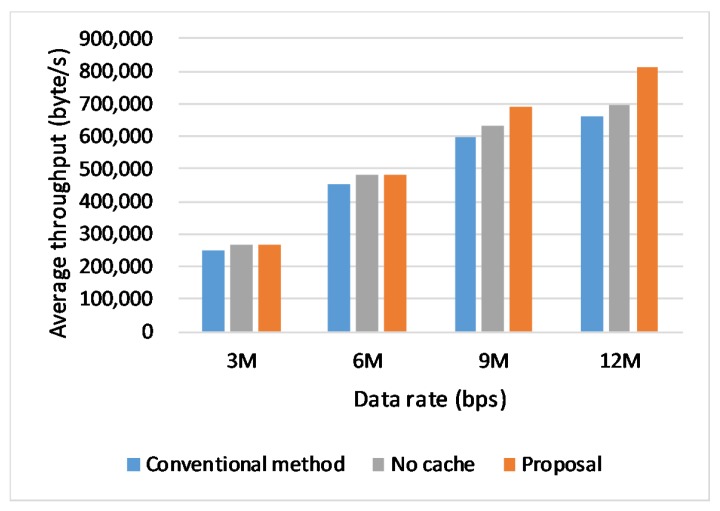
Throughput in different data rates.

The Figure 5 is a comparison of the transmission performance for different link rates. The vertical axis presents the transmission average throughput. The result shows that the proposed handover scheme always performs a higher throughput, compared with the conventional handover, according to the Figure 5. Especially, the result after adding a cache on the base station performs the best. When the link rate increases, our proposal shows more advantages by having a higher average throughput. So we can give a conclusion that our proposal benefits the network performance in high-speed networks. We also pick up the real-time throughput and CWND to further confirm the benefits that our proposal brings about. We select the transmission data at the rate of 9 Mbps for the further discussions.

**Figure 6:**
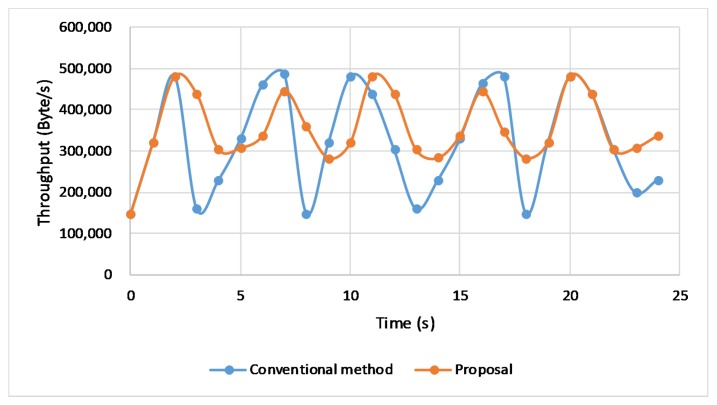
Real-time throughput.

The Figure 6 depicts the real-time throughput changes. We can clearly recognize the advantage of proposed handover scheme. It shows that throughput may plunge to the relatively small value when handover happens with the conventional method, leading to a lower average throughput. Besides, our proposal keeps the connection unchanged when handover happens offering more stable throughput, thus providing better performance.

**Figure 7:**
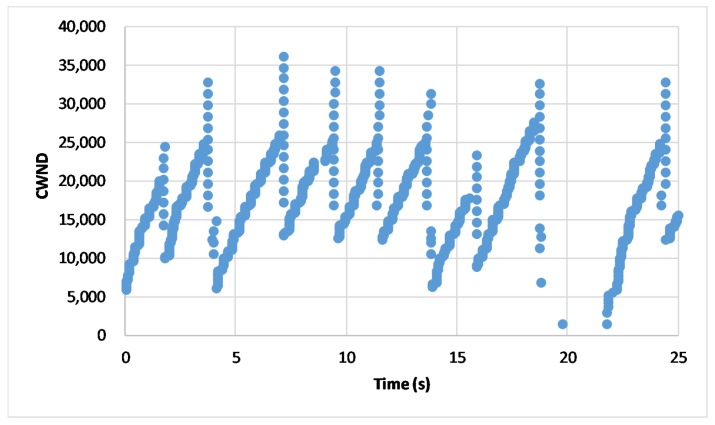
Real-time CWND in the proposed scheme.

**Figure 8:**
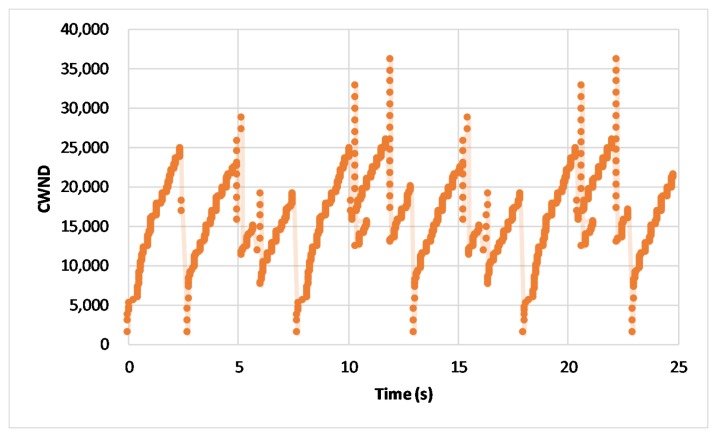
Real-time Congestion WiNDow (CWND) in the conventional approach.

The Figure 7 and Figure 8 depict a section of real-time change of CWND for 25 seconds in the proposed scheme and the conventional change method, respectively. Figure 7 shows the change of CWND in the proposed scheme. We can see that CWND always keeps the higher value, even if the handover happens. As shown in the Figure 8, compared with the proposed scheme, the conventional method would change the connection when a handover happens, so that the CWND would be suddenly initialized as the minimum value when the connection is re-established. As the CWND is the basis of transmission rate in transport layer, frequent initializations of CWND bring continuous sudden decrease of transmission rates, so that the network performance would be affected negatively.

### 6.3. Effect of Vehicle Velocities

**Figure 9:**
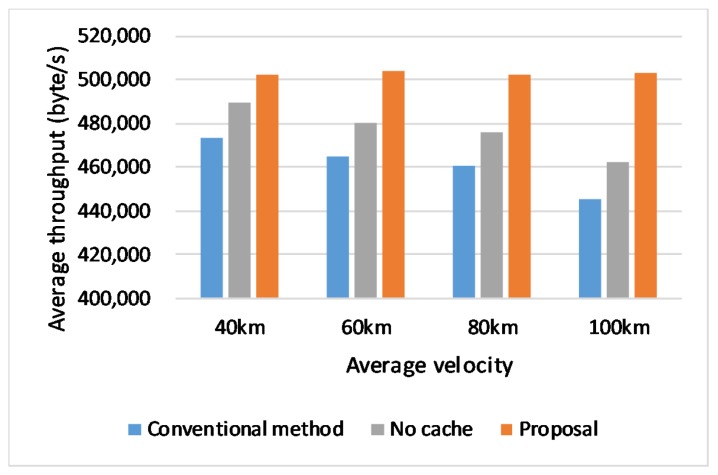
Throughput in different velocities.

In the second scenario, the vehicle’s velocities are set to 40, 60, 80, and 100 km/s, in order to compare the performances of our proposal and the conventional handover with different vehicle velocities. The link rate is set to 6 Mbps, and the result is shown in Figure 9. The result shows that when vehicle velocity becomes faster, the throughput of the proposal gradually decreases, but the performance of the change connection operation seems to be most affected by the change of velocity. The faster the velocity is, the lower the throughput becomes. As each simulation runs over the same map, the handover happening interval is influenced by the velocity. The fast speed means frequent handover, which makes network performance decrease. Thus the performance in the conventional operation is more affected by the vehicle velocity as the proposed scheme does.

**Figure 10:**
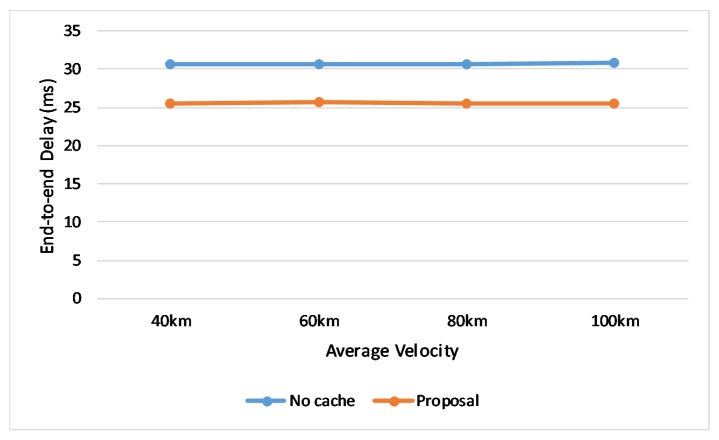
Comparison of end-to-end delay.

To evaluate how the caching scheme effect to the transmission delay, we also extract the delay of transmitting data from the “No cache” method and our proposal. The result from Figure 10 shows the certain decrease in transmission delay with the poroposed caching scheme. With the change of velocity, the value not change so much, because the result is average value from every single packet which is not that much influenced by the change in small range.

### 6.4. Effect of Vehicle Densities

**Figure 11:**
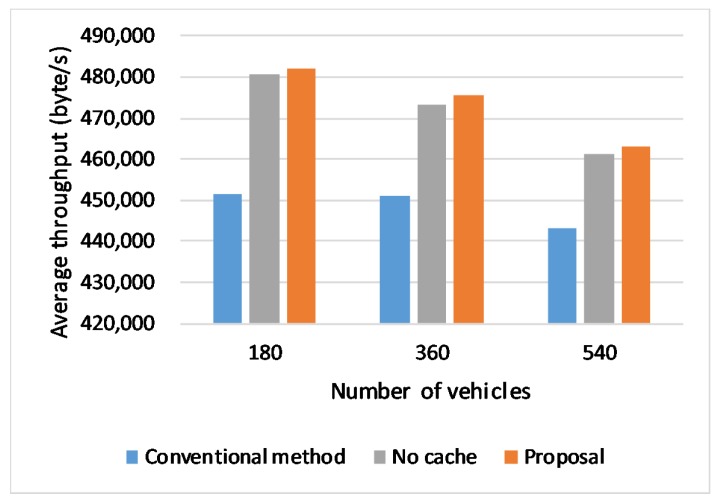
Throughput in different numbers of vehicles.

Figure 11 shows the throughput for different vehicle densities. As result shows, With the growth of the number of vehicles, the burden on the base station will increase, as well as data collision will happen, which affects the network performance. However, our proposal still keeps advantages over conventional handover. As the network environment getting worse, our caching scheme exerts its strengths.

### 6.5. Effect of Beacon Intervals

**Figure 12:**
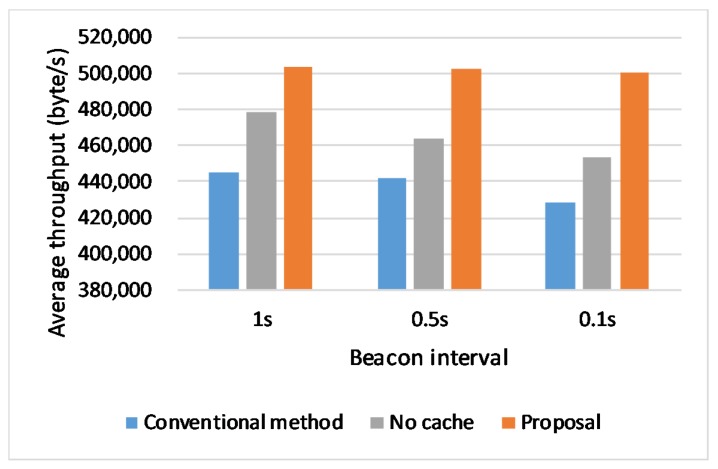
Throughput in different Beacon intervals.

The forth scenario assumes that vehicle runs in the context of different beacon intervals. We keep the link rate at 6 Mbps and speed in 60 km/s. To evaluate the performance influenced by the background traffic, we set different beacon interval values (beacon interval = 0.1 s, 0.5 s, 1 s) for different simulation runs. The simulation result is shown in Figure 12 and indicates that whatever the time interval is, the proposal performs better than the conventional method when a handover happens. Especially, it is obvious that the proposed scheme has greater advantages when the handover happens more frequently.

### 6.6. Effect of Background Noise Levels

**Figure 13:**
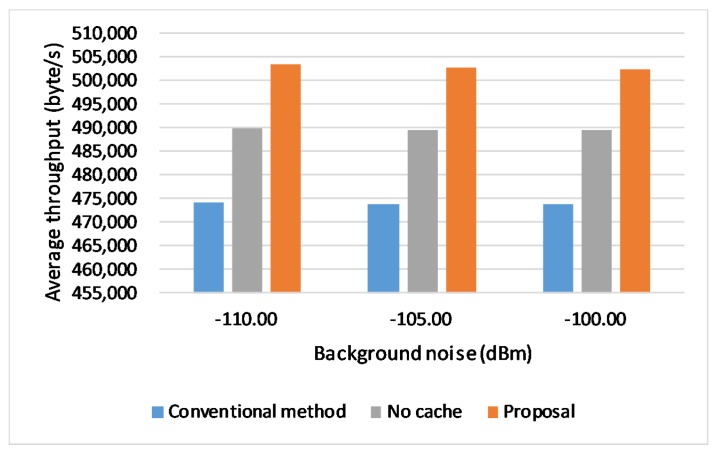
Throughput in different background noise levels.

We also evaluate the proposed scheme in different background noise levels. We set different background noise value as −110, −105, −100 dBm for the different set of simulations and kept link rate at 6 Mbps. The result is shown in Figure 13. With the background noise increases the performance of the conventional handover method does not seem to be influenced that much. Due to the system overhead and limited computing capability, traffic congestion is more likely to happen when the vehicle moves in a bad communication environment. When a handover happens with the conventional method (change connection operation), the changing trend of CWND is the same as the one in a serious traffic congestion according to the congestion control algorithm. Therefore, the performance of the conventional method is not that much influenced by the vehicle velocity as the proposal does.

### 6.7. Effect of Distance between Base Stations

**Figure 14:**
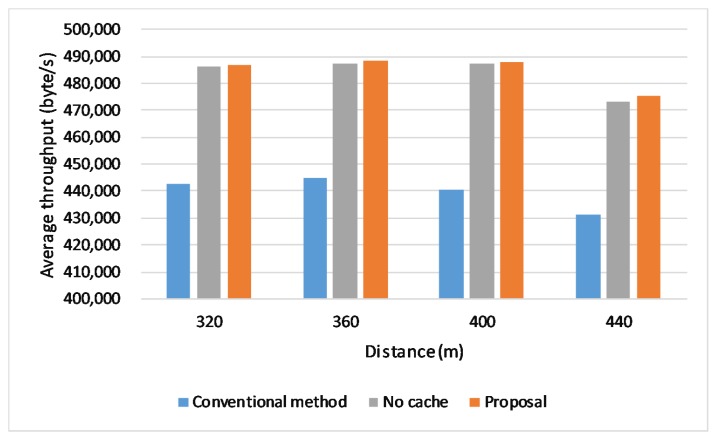
Throughput in different distances between base stations.

We evaluate the proposed scheme with different distances between the base stations. The result is shown in Figure 14. The proposed scheme shows its advantages no matter how long the distance is. When the distances between base stations are not too far, the performance is getting better as the distances become longer because the handover frequency is getting lower. However, when the distances become longer, the network performance is not only influenced by the handover frequency, but also affected by the external factors such as the signal quality, resulting in a lower average throughput.

## 7. Conclusions

We proposed a SDN-based handover scheme that was built on a two-level SDN controller architecture. By employing an SDN-based approach which achieves a global view of vehicles, the proposed scheme could execute handover proactively and forward the data in advance to the new base station in a handover process, which could significantly reduce the handover latency and efficiency. The proposed scheme also uses a caching approach at the base stations to minimize the package loss and transmission delay caused by the handover. We conducted exhaustive simulations in different network environments by changing the vehicle velocity, link rates and wireless channel conditions. The results showed that the proposed scheme achieved better network performance than existing baseline approaches, especially in a scenario with frequent topology changes.

## Figures and Tables

**Table 1:** Simulation parameters.

Parameters	Values
Routing Protocol	AODV
Transport Layer	TCP(RENO)
Interface	IEEE 802.11p
Number of Vehicles	180, 360, 540
Average velocity	40 km, 60 km, 80 km, 100 km
Data Rate	3 Mbps, 6 Mbps, 9 Mbps, 12 Mbps
Beacon Interval	1 s, 0.5 s, 0.1 s
Simulation Topology	Grid and Straight road
Topology Size	1000 m × 600 m, 2000 m with 4 lanes
